# Targeting promiscuous heterodimerization overcomes innate resistance to ERBB2 dimerization inhibitors in breast cancer

**DOI:** 10.1186/s13058-019-1127-y

**Published:** 2019-03-21

**Authors:** Sean P. Kennedy, Jeremy Z. R. Han, Neil Portman, Max Nobis, Jordan F. Hastings, Kendelle J. Murphy, Sharissa L. Latham, Antonia L. Cadell, Dushan Miladinovic, Gabriella R. Marriott, Yolande E. I. O’Donnell, Robert F. Shearer, James T. Williams, Amaya Garcia Munoz, Thomas R. Cox, D. Neil Watkins, Darren N. Saunders, Paul Timpson, Elgene Lim, Walter Kolch, David R. Croucher

**Affiliations:** 1 The Kinghorn Cancer Centre, Garvan Institute of Medical Research, 370 Victoria St, Darlinghurst, Sydney, NSW 2010 Australia; 2 Systems Biology Ireland, University College Dublin, Belfield, Dublin 4 Ireland; 3 St Vincent’s Hospital Clinical School, University of New South Wales, Sydney, NSW 2052 Australia; 4 School of Medicine, University of Notre Dame, Sydney, NSW 2011 Australia; 5 School of Medical Sciences, University of New South Wales, Sydney, NSW 2025 Australia; 6 Conway Institute of Biomolecular and Biomedical Research, University College Dublin, Belfield, Dublin 4 Ireland; 7 School of Medicine and Medical Science, University College Dublin, Belfield, Dublin 4 Ireland

**Keywords:** ERBB2, Pertuzumab, Receptor tyrosine kinases, Breast cancer, Heterodimers

## Abstract

**Background:**

The oncogenic receptor tyrosine kinase (RTK) ERBB2 is known to dimerize with other EGFR family members, particularly ERBB3, through which it potently activates PI3K signalling. Antibody-mediated inhibition of this ERBB2/ERBB3/PI3K axis has been a cornerstone of treatment for ERBB2-amplified breast cancer patients for two decades. However, the lack of response and the rapid onset of relapse in many patients now question the assumption that the ERBB2/ERBB3 heterodimer is the sole relevant effector target of these therapies.

**Methods:**

Through a systematic protein-protein interaction screen, we have identified and validated alternative RTKs that interact with ERBB2. Using quantitative readouts of signalling pathway activation and cell proliferation, we have examined their influence upon the mechanism of trastuzumab- and pertuzumab-mediated inhibition of cell growth in ERBB2-amplified breast cancer cell lines and a patient-derived xenograft model.

**Results:**

We now demonstrate that inactivation of ERBB3/PI3K by these therapeutic antibodies is insufficient to inhibit the growth of ERBB2-amplified breast cancer cells. Instead, we show extensive promiscuity between ERBB2 and an array of RTKs from outside of the EGFR family. Paradoxically, pertuzumab also acts as an artificial ligand to promote ERBB2 activation and ERK signalling, through allosteric activation by a subset of these non-canonical RTKs. However, this unexpected activation mechanism also increases the sensitivity of the receptor network to the ERBB2 kinase inhibitor lapatinib, which in combination with pertuzumab, displays a synergistic effect in single-agent resistant cell lines and PDX models.

**Conclusions:**

The interaction of ERBB2 with a number of non-canonical RTKs activates a compensatory signalling response following treatment with pertuzumab, although a counter-intuitive combination of ERBB2 antibody therapy and a kinase inhibitor can overcome this innate therapeutic resistance.

**Electronic supplementary material:**

The online version of this article (10.1186/s13058-019-1127-y) contains supplementary material, which is available to authorized users.

## Background

ERBB2 (HER2) is a receptor tyrosine kinase (RTK) and potent oncogene that is amplified in ~ 20% of breast cancer cases. Amplification of ERBB2 is known to be associated with an aggressive tumour phenotype, shorter disease-free survival and poor overall survival [1, 2]. The prognosis for patients diagnosed with ERBB2-amplified (ERBB2+) breast cancer has improved since the introduction of monoclonal antibody therapy in both the adjuvant and metastatic setting. The standard of care for early-stage ERBB2+ breast cancer is chemotherapy plus trastuzumab (Herceptin™). Trastuzumab is a humanised monoclonal antibody that binds to the extracellular domain IV of ERBB2 thereby inhibiting ligand-independent hetero-dimerization between ERBB2 and other EGFR family members and also eliciting an antibody-mediated, host-directed cytotoxic response [3]. However, while trastuzumab chemotherapy combinations are effective in improving survival in early-stage ERBB2+ breast cancer, approximately 25% of patients will relapse within 10 years [4].

To counter this resistance, a new generation of ERBB2-targeting therapies has been developed, including pertuzumab (Perjeta™), a domain II targeting antibody specifically designed to inhibit ligand-dependent ERBB2 hetero-dimerization [5]. When included in the first-line therapy with trastuzumab and chemotherapy, pertuzumab demonstrated a clinically meaningful improved survival for patients with metastatic ERBB2+ breast cancer and a smaller survival benefit in the adjuvant setting [6, 7]. This dual ERBB2 antibody combination has also improved pathological complete response (pCR) rates compared with single ERBB2 antibodies plus chemotherapy [8]. Based on these pivotal clinical trial results, this combination is now FDA approved for use in both early-stage neoadjuvant and adjuvant, and metastatic ERBB2+ breast cancer.

Another ERBB2-directed therapy mature in clinical development is lapatinib, an oral small molecule dual tyrosine kinase inhibitor (TKI) of EGFR and ERBB2. Lapatinib is FDA approved for use in combination with capecitabine for patients with advanced breast cancer who have progressed on trastuzumab and chemotherapy [9]. It has also been shown to improve pCR rates in combination with trastuzumab compared with trastuzumab alone [10]. Interestingly, all of the dual ERBB2-targeting therapies evaluated have focused primarily on trastuzumab combinations, while pertuzumab and lapatinib have been used as partnering agents to trastuzumab rather than as a combination in its own right.

In spite of this progress, the identification of patients that will benefit from ERBB2-directed therapies is greatly hindered by a lack of biomarkers for predicting therapeutic response. This is despite the extensive retrospective analysis of phase III clinical trial data for the potential roles of related RTKs (i.e. EGFR, ERBB3, IGF-1R), ligands (i.e. EGF, TGFα) or PI3K pathway alterations [11, 12]. Therefore, the expression of ERBB2 itself is currently the only biomarker to guide treatment decisions within this patient cohort.

As it lacks an extracellular ligand, ERBB2 is thought to be activated by hetero-dimerization with other ligand-activated members of the EGFR family (i.e. EGFR, ERBB3 and ERBB4) [13]. Physiologically, ERBB2 is unable to form a homodimer and exists either solely as a monomer or heterodimer with other ligand-bound family members [14]. However, in ERBB2+ breast cancer, the highly elevated levels of ERBB2 lead to a violation of these physiological constraints and the formation of both ERBB2 homodimers and ligand-independent heterodimers [14, 15]. The ERBB2:ERBB3 heterodimer is considered to be the main signalling entity through which ERBB2 mediates its oncogenic function, chiefly through potent activation of PI3K/Akt signalling [16, 17].

Since the discovery of dimerization between EGFR family members [18–20], it was believed that these hetero-interactions occur exclusively within the EGFR family. However, an increasing number of studies now suggests that ERBB2, and indeed the other EGFR family members, is capable of more promiscuous behaviour than was originally appreciated [21].

To this end, we have now used a bimolecular fluorescence complementation screening platform, coupled with high-content imaging, to systematically evaluate the ability of ERBB2 to interact with a wide array of RTKs from outside the EGFR family. We also report that while pertuzumab significantly inhibits the activity of the ERBB3/PI3K signalling axis, it paradoxically promotes the activation of non-canonical heterodimers and limits its own efficacy through ERBB2-mediated ERK activation. Additionally, we also demonstrate that this network of unexpected heterodimers can be targeted by dual therapy with pertuzumab and lapatinib.

Unlike the combination of trastuzumab with either pertuzumab or lapatinib, the joint action of pertuzumab plus lapatinib is capable of eliciting a synergistic effect in both cell lines and patient-derived xenograft (PDX) models resistant to each individual antibody. Despite extensive clinical trials evaluating various permutations of ERBB2-targeting drugs [22], this combination has yet to undergo clinical trials. Therefore, this promising approach may provide additional avenues for increasing the efficacy of ERBB2-targeted therapies in patients resistant to single-agent therapy and also further options for developing biomarkers to predict the individual patient response.

## Methods

### Antibodies, plasmids and reagents

The C-terminal GFP monoclonal antibody (11814460001) was from Roche, and N-terminal GFP monoclonal antibody (MMS-118P) was from Covance. Antibodies against total ERK (#9201), phospho-ERK^T202/Y204^ (#4370), total P38 (#8690), phospho-P38^T180/Y182^ (#4511), total JNK (#9258), phospho-JNK^T183/Y185^ (#9251), total AKT (#9272), phospho-AKT ^S473^ (#4060), total STAT3 (#9139), phospho-STAT3 (#9131), total PLCg (#5690), phospho-PLCg (#14008), total ERBB2 (#2165), phospho-ERBB2 Y1248 (#22475), phospho-ERBB2 Y1221/1222 (#2243), total EGFR (#4267), total ERBB3 (#12780), phospho-ERBB3 (#2842) total MET (#8198), total NTRK1 (#2508), phospho-NTRK1 (#4621), total TIE-2 (#7403) and pTIE-2 (#4221) and PY100 (#9411) were from Cell Signaling (MA, USA). Antibodies against phospho-MET^Y1234^, Grb2 (sc-8034) and IGF-1R (sc-731) were from Santa Cruz Biotechnology (TX, USA). Antibodies against total MERTK (Y323) and ERBB2 (CB11) were from Abcam (MA, USA). The SHC antibody was from Calbiochem (ST1033). The Ki67 (SP6) antibody was from ThermoFisher Scientific (RM-9106-S1). The actin monoclonal antibody (AC-15) was from Sigma-Aldrich (MO, USA). The therapeutic antibody pertuzumab (Perjeta®) was obtained from Genentech (CA, USA). Trastuzumab (Herceptin®) was a kind gift from Dr Norma O’Donovan (National Institute for Cellular Biotechnology, Dublin, Ireland).

pDEST-V1 and pDEST-V2 vectors were assembled from various recombinant and synthetic components, as previously described [23]. All donor plasmid vectors containing full-length RTKs (Additional file 1: Table S1) were obtained from Addgene (MA, USA), DNASU (AZ, USA), Genomecube (Source Bioscience, Nottingham, UK) or Genecopoeia (MD, USA). All pDONR223 RTK constructs (Addgene) were a kind gift from Dr William Hahn and Dr David Root [24]. Vectors expressing V1- or V2-labelled RTK fusions were generated by recombination cloning into pDEST-V1 or pDEST-V2 destination vectors using Gateway LR Clonase enzyme mix (Life Technologies) according to manufacturer’s instructions and sequence verified. Where necessary, stop codons were removed using the QuikChange II Site-Directed Mutagenesis Kit (Agilent Technologies, CA, USA). An expression vector encoding full-length Venus fluorescent protein was a kind gift from Dr Stephen Michnick (University of Montreal). The pLentiCMV Puro DEST ERKKTRClover was a gift from Markus Covert (Addgene plasmid # 59150). Plasmid transfection was performed using JetPRIME (Polyplus Transfection) according to the manufacturer’s instructions.

The multi TKIs cabozantinib (Cabometyx®) and BMS-777607 were obtained from Selleckchem (NSW, Australia). Lapatinib ditosylate was from Santa Cruz Biotechnology (TX, USA). CellTiter96 Aqueous Non-Radioactive Cell Proliferation Assay (MTS) reagents were from Promega (WI, USA).

### Cell lines

The HEK293T cell line was cultured in DMEM containing 10% FCS under standard tissue culture conditions (5% CO_2_, 20% O_2_). All breast cancer cell lines were obtained from the American Type Culture Collection, except for the MDA-MB-231 and T-47D lines (EG&G Mason Research Institute, Worcester, MA, USA) and MCF-7 line (Michigan Cancer Foundation, Rochester, MI, USA). Cell lines were authenticated by short tandem repeat polymorphism, single-nucleotide polymorphism, and fingerprint analyses, passaged for less than 6 months and cultured as previously described [25]. Stable cell lines expressing the ERK-KTR biosensor were generated by lentiviral transduction as previously described [26]. Cytotoxicity assays were performed using the CellTiter 96 Aqueous Non-Radioactive Cell Proliferation Assay, according to the manufacturer’s instructions (Promega). Calculations of synergy were performed using CompuSyn (Version 1.0).

### Western blotting and immunoprecipitation

Lysates for western blotting and immunoprecipitation were prepared using the normal lysis buffer (50 mM Tris HCl pH 7.4, 150 mM NaCl, 1 mM EDTA, 1% (*v*/*v*) Triton X-100) containing protease inhibitor cocktail (p8340, Sigma) and 0.2 mM sodium orthovanadate. Immunoprecipitation was performed using protein-A/G agarose beads (Invitrogen), as previously described [27]. SDS-PAGE electrophoresis and western blotting were performed using the NuPAGE SDS PAGE Gel System and NuPAGE Bis Tris Precast Gels (4–12%) (Life Technologies). Western Lightning PLUS Enhanced Chemiluminescent Substrate (PerkinElmer) was for imaging western blots on the Vilber Lourmat Fusion chemiluminescent imaging system. Quantitative western blotting was performed using multistrip western blotting [28]. The Human Phospho-Kinase Antibody Array was obtained from R&D Systems (MN, USA) and used according to manufacturer’s instructions.

### Bimolecular fluorescent complementation and high-content imaging

To investigate receptor interactions with ERBB2 by BiFC, we utilised the pDEST-ERBB2-V1 plasmid as the ‘bait’ in all experiments. When co-transfected with a complementary pDEST-V2 plasmid containing another RTK (Additional file 1: Table S1), a positive interaction could be determined by the observation of a fluorescent signal from the recombined Venus protein [23].

HEK-293 T cells were cultured in glass-bottomed, black 96-well plates (5000 per well) and transfected with both 10 ng of pDEST-ERBB2-V1 and 10 ng of pDEST-V2 containing another RTK. The cells were incubated under standard conditions for 20 h before the addition of Hoechst 33432 (1 μg/mL) for 15 min before visualisation and quantification using the Cellomics ArrayScan (ThermoScientific). High-content imaging was performed using the nuclear translocation function of the HCS Studio Cell Analysis Software. Briefly, cells were identified and filtered by nuclear staining (Hoechst 33432). A nuclear mask was generated for each individual nucleus, and the BiFC signal quantified within a 4-pixel cytoplasmic ring. Measurements for each receptor pairing were taken across 20 fields in 3 wells, averaging between 5000 and 10,000 cells.

### Confocal microscopy

For visualisation of BiFC receptor interactions, HEK-293 T cells grown on glass coverslips were transfected with 500 ng of each BiFC construct and incubated for 16–24 h under standard tissue culture conditions. Cells were incubated with Hoechst 33342 for 5 min, followed by fixation with 1% para-formaldehyde and mounting with Mowiol mounting media. Images were collected using the Leica DMI 6000 SP8 confocal microscope.

Proximity-mediated ligation assay (PLA) was performed on cell lines using the Duolink® In Situ Red Starter Kit (Sigma-Aldrich, St Louis, USA), according to manufacturer’s instructions. Images were collected using the Leica DMI 6000 SP8 confocal microscope.

### Immunohistochemistry

Immunohistochemistry was performed on formalin-fixed paraffin-embedded sections using the Leica BOND RX (Leica, Wetzlar, Germany). Slides were first dewaxed and rehydrated. Heat-induced antigen retrieval was performed with either citrate pH 6 (ERBB2) or EDTA pH 9 (ERBB3, MET, MERTK, TIE2, NTRK1, Ki67) retrieval buffer for 20 min at 100 °C (ERBB2, ERBB3, MET, MERTK, TIE2, NTRK1) or 40 min at 92 °C (Ki67). Primary antibodies were diluted 1:100 (ERBB2, ERBB3, MET, MERTK, TIE2, NTRK1) or 1:500 (Ki67) in Leica antibody diluent and incubated for 60 min on slides. Antibody staining was completed using the Bond Polymer Refine IHC protocol and reagents (Leica, Wetzlar, Germany). Slides were counterstained on the Leica Autostainer XL (Leica, Wetzlar, Germany). Coverslips were placed with the Leica CV5030 glass coverslipper (Leica, Wetzlar, Germany), and brightfield images were taken on the Aperio CS2 slide scanner (Leica, Wetzlar, Germany). Quantification of Ki67 staining was performed on three fields of view for each tumour section and quantified using the particle analysis function of ImageJ (v1.49).

PLA was performed on formalin-fixed paraffin-embedded tissue sections using the Duolink® In Situ Red Starter Kit with the following modifications. Slides were first dewaxed and rehydrated (Leica Autostainer XL, Leica, Wetzlar, Germany). Antigen retrieval was performed on slides in a citrate buffer (pH 6) by incubation at 95 °C for 40 min (Leica Bond RX, Leica, Wetzlar, Germany). Slides were washed twice in 200 mL of TBS (150 mM NaCl, 10 mM Tris pH 7.4) containing four drops of 1 M glycine and incubated at room temperature for 5 min, before a final 5-min wash in TBS. PLA steps were performed in a humidity chamber unless specified otherwise. Samples were encircled with a hydrophobic pen and then blocked in Duolink® blocking buffer for 30 min–1 h at 37 °C. Primary antibodies were made up 1:100 in Duolink® antibody diluent and incubated overnight at 4 °C. Anti-rabbit plus and anti-mouse minus PLA probes were added and incubated for 2 h. Samples were incubated with ligation stock diluted 1:5 in high-purity water with 1:40 ligase for 1 h at 37 °C to allow oligo hybridisation and ligation. The resulting circular DNA strand was then amplified overnight at 37 °C with amplification stock diluted 1:5 in high-purity water with 1:80 polymerase. Tissue was then stained with DAPI diluted 1:50 in high-purity water for 15 min at room temperature. Samples were air dried then mounted using Vectashield® Mounting Medium (Vector Laboratories, Burlingame, USA). The PLA signal was excited at 594 nm and detected at 624 nm and imaged on the Leica DMI 6000 SP8 Advanced laser scanning confocal microscope (Leica, Wetzlar, Germany).

### Patient-derived xenograft models

The generation and maintenance of PDX HCI-012 have been described previously [29]. It was derived from the pleural effusion of a patient with metastatic ER-negative, PR-negative and ERBB2-amplified breast cancer and established in immunocompromised mice. All in vivo experiments, procedures and endpoints were approved by the Garvan Institute of Medical Research Animal Ethics Committee. Four-cubic millimetre sections of tumour tissue from a fresh passage were implanted into the fourth inguinal mammary gland of female NOD-SCID-IL2γR−/− (NSG) mice (Australian BioResources Pty Ltd), as previously described [30]. Tumour growth was assessed twice weekly by a calliper measurement, and mice were randomised to treatment arms when tumours reached 150–250 mm^3^ (using the formula: width^2^ × length × 0.5). Pertuzumab was administered by intraperitoneal injection at an initial dose of 12 mg/kg with subsequent weekly injections of 6 mg/kg. Lapatinib was administered at 50 mg/kg 5 days per week by oral gavage. Mice were treated over a period of 21 days or until tumour volume reached 1000 mm^3^. Growth rates (mm^3^ per day) were determined by linear regression (*R*^2^ vehicle 0.99, lapatinib 0.98, pertuzumab 0.97, combination 0.99) and compared using the one-way ANOVA and *T* tests of each pairwise combination with Tukey’s correction for multiple testing. All statistical analyses were performed using built-in functions in GraphPad Prism (Version 7, GraphPad Software).

## Results

### Efficacy of ERBB2-targeting monoclonal antibodies

**Fig. 1:**
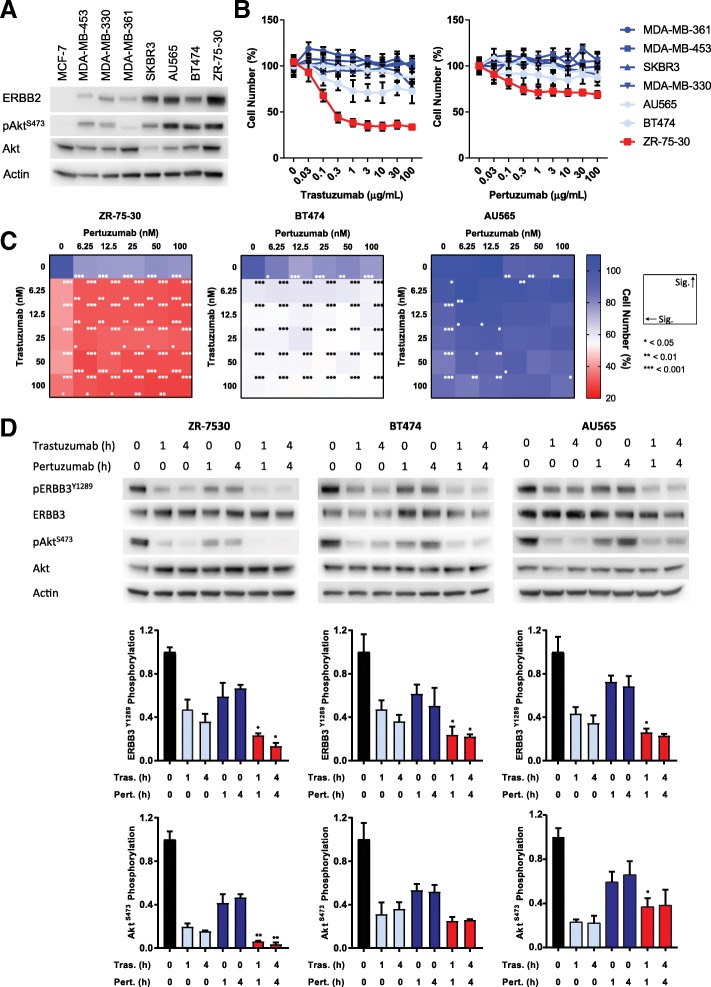
Targeting ERBB2 with therapeutic monoclonal antibodies. **a** Western blotting showing the expression of ERBB2, pAkt^S473^ and Akt in a panel of breast cancer cell lines. **b** Cell viability assays performed on the panel of lines with trastuzumab and pertuzumab at the concentrations indicated for 5 days (*n* = 6, mean ± SD). **c** Combinatorial cell viability assays with trastuzumab and pertuzumab at the concentrations indicated for 5 days (*n* = 6, mean). Raw data is presented in Additional file 2: Figure S1B. **d** Western blotting showing the phosphorylation of ERBB3^Y1289^ and Akt^S473^ following treatment with trastuzumab (100 nM), pertuzumab (100 nM) and the combination of both, at the time points indicated. Blots were re-probed with an actin antibody for quantification. A representative image is shown from three independent replicates (*n* = 3, mean ± SD). All drug treatments were significantly different from control. For simplicity, the indicated significance compares the combination to trastuzumab only (*p < 0.05, **p < 0.01)

Both trastuzumab and pertuzumab are expected to have significant, cell-autonomous efficacy against ERBB2+ breast cancer cell lines, based upon their respective action against either ligand-independent or ligand-dependent ERBB2 signalling. However, within a panel of ERBB2+ breast cancer cell lines (Fig. 1a, Additional file 2: Figure S1A), trastuzumab only efficiently inhibited the growth of the high ERBB2-expressing ZR-75-30 cell line (Fig. 1b, IC_50_ 0.2 μg/mL, ~ 1.3 nM). Trastuzumab also partly inhibited the growth of the other high ERBB2-expressing lines, BT474, AU565 and SKBR3, but only at high concentrations for the latter two. Pertuzumab was even less effective, only inhibiting the growth of the ZR-75-30 line by ~ 30%, BT474 by ~ 20% and AU565 and SKBR3 by ~ 10% (Fig. 1b).

However, as these drugs are not often used as single agents in the clinical setting, we also investigated their combined effect on these high ERBB2-expressing cell lines (Fig. 1c). In line with their activity as single agents, trastuzumab and pertuzumab were both effective towards the ZR-75-30 line and also displayed an additive effect when present in combination (Fig. 1c, Additional file 2: Figure S1B). However, the relatively small effect of both drugs was not additive for either the BT474 or AU565 lines.

Because of their complementary inhibitory mechanism, it is commonly held that the combination of trastuzumab and pertuzumab will have increased efficacy by entirely blocking the signalling capacity of ERBB2 [3]. However, our findings demonstrate that combination therapy with these two drugs may not always be beneficial, even within the context of high ERBB2 expression. As would be expected, each of these high ERBB2-expressing cell lines also displayed elevated levels of phosphorylated Akt (Fig. 1a). We therefore investigated the ability of these drugs individually, and in combination, to inhibit ERBB3 and Akt activation, as this is considered the main signalling pathway through which ERBB2 mediates its oncogenic function [16]. Consistent with these expectations, trastuzumab inhibited ERBB3 phosphorylation by ~ 60% and Akt phosphorylation by 60–80% in all cell lines (Fig. 1d), while pertuzumab inhibited ERBB3 phosphorylation by 30–50% and Akt phosphorylation by 40–60%. The combination of trastuzumab and pertuzumab had a significantly additive effect upon ERBB3 phosphorylation in all three cell lines. However, this was not the case for downstream signalling to Akt, for which this combination only displayed an additive effect in the ZR-75-30 line.

As trastuzumab is proposed to inhibit ligand-independent dimerization between ERBB2 and ERBB3, this appears to be the predominant mechanism of ERBB2-mediated ERBB3 activation in these ERBB2+ cell lines. Although more importantly, these findings clearly demonstrate that there is a disconnection between the ability of ERBB2-targeting therapeutic antibodies to inhibit the ERBB2/ERBB3/PI3K signalling axis and their ability to exert an influence upon cell growth. This further suggests that our understanding of ERBB2 dimerization and signalling in this pathological context is incomplete and that targeting ERBB2 in this manner will not always be sufficient to completely inhibit its oncogenic function.

### ERBB2 interacts with a wide array of RTKs

**Fig. 2:**
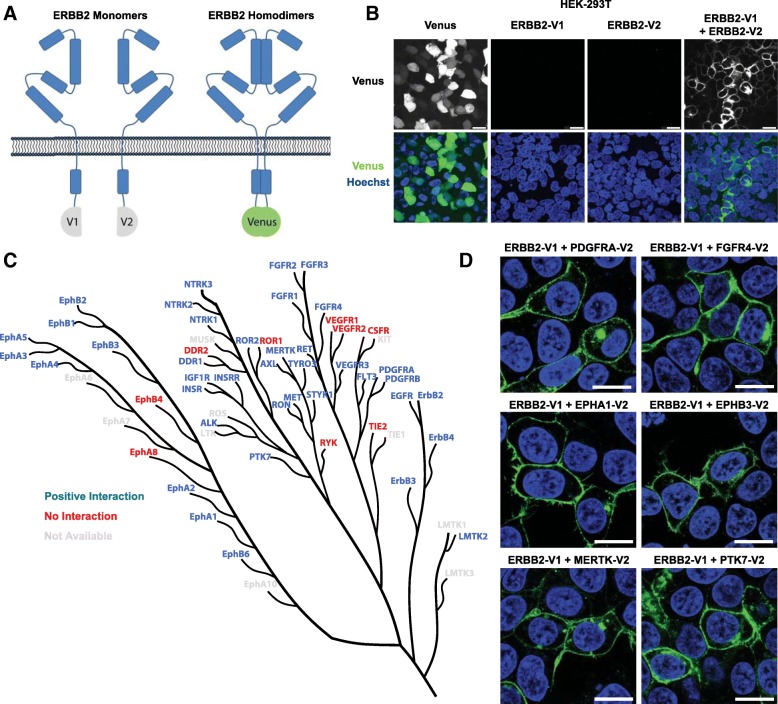
ERBB2 interaction screen. **a** Schematic of the BiFC assay used to visualise ERBB2 dimerization. **b** Confocal fluorescence microscopy of HEK-293 T cells following transfection with plasmids containing a full-length Venus control, ERBB2-V1, ERBB2-V2 or co-transfected with ERBB2-V1 and ERBB2-V2 (scale bar = 25 μm). **c** Dendrogram showing the sequence-based relationship between all 61 RTKs and their ERBB2 interaction status detected using the BiFC assay and high-content imaging. The dendrogram was adapted from Manning et al. 2002 (24). **d** Confocal fluorescence microscopy of HEK-293 T cells following transfection with plasmids containing ERBB2-V1 and the V2 tagged RTKs indicated (scale bar = 15 μm)

Therefore, to investigate the potential for ERBB2 to form heterodimers across the full spectrum of RTK families, we utilised a bimolecular fluorescence interaction technique that we and others have previously used to visualise the formation of EGFR family dimers [23, 31]. This technique uses the individual N-terminal (V1) and C-terminal (V2) fragments of the Venus fluorescent protein, which are non-fluorescent and associate with low affinity in the absence of an interaction between fusion partners. However, their close co-localisation upon interaction of bait and prey fusion proteins favours refolding of these split domains into a functional β-barrel structure containing the fluorophore [32] (Fig. 2a). Using this technique, a membranous fluorescent signal can be observed following co-transfection of ERBB2-V1 and ERBB2-V2 into HEK-293 T cells, but not for the individually tagged constructs in isolation (Fig. 2b, Additional file 2: Figure S2A). We have also previously observed this for the formation of ERBB2:EGFR and ERBB2:ERBB3 heterodimers [23], and here we have adapted this technique to screen for the formation of heterodimers between ERBB2 and a library of 46 full-length RTKs (Fig. 2c) (Additional file 1: Table S1).

Following co-transfection of the ERBB2-V1 plasmid with each of the 46 V2-tagged RTKs, we used high-content imaging to detect a fluorescent signal from the refolded Venus protein and filtered this for significance over the background signal generated by a non-interacting pair, ERBB2-V1 and Histone 2B-V2 (H2B-V2) (Additional file 2: Figure S2B). This interaction data was then overlaid onto an RTK dendrogram [33] in order to observe any phylogenetic patterns underlying heterodimer formation (Fig. 2c).

This library screen identified 37 RTKs that robustly dimerized with ERBB2 under these conditions, including all EGFR family members and other previously identified interacting partners, including IGF-1R, MET, AXL and NTRK1 [21]. However, many other novel interacting partners were also detected, including receptor families known to play a role in breast cancer, such as FGFRs, NTRKs and PDGFRs [34]. Interestingly, only a small number of non-interacting receptors clustered together, including VEGFR1 and VEGFR2, and EPHB4 and EPHA8.

Analysis of a subset of these unexpected interactions by confocal fluorescent microscopy confirmed that these non-canonical heterodimers are correctly localised at the plasma membrane (Fig. 2d). Therefore, these data suggest that ERBB2 may be able to exert its oncogenic activity through interaction with a broad array of RTKs, potentially diversifying the signalling response downstream of ERBB2 and also influencing the cellular response to ERBB2-targeting drugs.

### Pertuzumab increases tyrosine phosphorylation of alternative RTKs

**Fig. 3:**
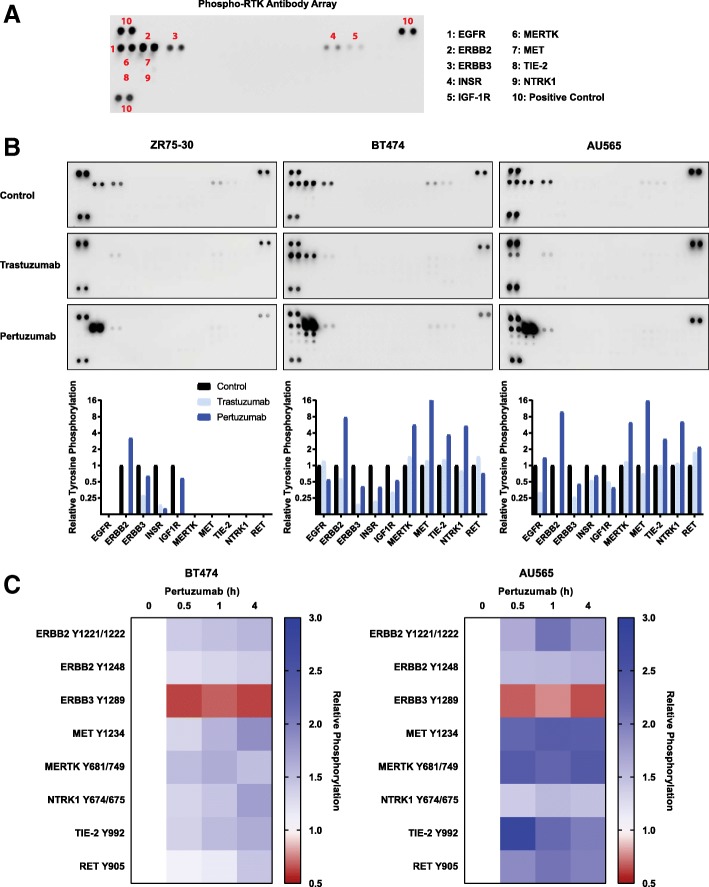
Analysis of RTK activation. **a** An example phospho-RTK antibody array showing the location of relevant RTKs within the array. **b** Phospho-RTK arrays developed using lysates from ZR-75-30, BT474 and AU565 cells treated with either trastuzumab (100 nM, 4 h) or pertuzumab (100 nM, 4 h). **c** AU565 and BT474 cells were treated with pertuzumab (100 nM) for the time points indicated. Western blotting was performed with lysates from these cells with the total and phospho-RTK antibodies indicated. Data is presented as a heat map showing the relative changes in phosphorylation (*n* = 3, mean), raw data and statistical analysis is presented in Additional file 2: Figure S4

While pertuzumab has been shown to inhibit the ligand-induced interaction between transfected ERBB2 and ERBB3 in COS-7 cells [5] and endogenous receptors in MCF-7 and SKBR3 cells [35], the influence of pertuzumab or trastuzumab upon the promiscuous signalling activity of ERBB2 is not well established. To investigate this, we utilised antibody-based arrays (Fig. 3a) to measure the relative tyrosine phosphorylation of 49 RTKs in the ZR-75-30, BT474 and AU565 cell lines following treatment with pertuzumab or trastuzumab (Fig. 3b). Under control conditions, tyrosine phosphorylation of EGFR, ERBB2, ERBB3, INSR and IGF-1R could be observed in all lines, apart from EGFR which is not expressed in the ZR-75-30 line (Additional file 2: Figure S1A). Tyrosine phosphorylation of ERBB3, IGF-1R and INSR decreased in all lines following treatment with either trastuzumab or pertuzumab, while trastuzumab decreased EGFR phosphorylation in the AU565 cells. However, tyrosine phosphorylation of ERBB2 actually increased following pertuzumab treatment in all lines, as did phosphorylation of a subset of other RTKs, including MERTK, MET and NTRK1 in both AU565 and BT474 lines, and TIE-2 and RET in AU565 cells (Fig. 3b). Notably, the ZR-75-30 line expresses either low or undetectable levels of MET, MERTK and NTRK1 (Additional file 2: Figure S1A), which likely underlies the lack of phosphorylation of these receptors in this cell line.

The increased tyrosine phosphorylation of ERBB2 was validated by immuno-precipitation and blotting with PY100 (Additional file 2: Figure S3A). However, the decreased phosphorylation of ERBB2 following trastuzumab treatment on the antibody array is likely an artefact of antibody competition, as this decrease was not observed by immunoprecipitation and blotting with PY100 (Additional file 2: Figure S3B).

To confirm the results from the antibody array, we also undertook western blotting with phospho-specific antibodies (Fig. 3c, Additional file 2: Figure S4). This analysis also demonstrated that pertuzumab significantly decreased ERBB3^Y1289^ phosphorylation in both AU565 and BT474 lines, but also significantly increased the phosphorylation of ERBB2^Y1221/1222^, ERBB2^Y1248^, MET^Y1234^, NTRK1^Y674/Y675^, MERTK^Y681/Y749^, TIE-2^Y992^ and RET^Y905^ in both lines.

**Fig. 4:**
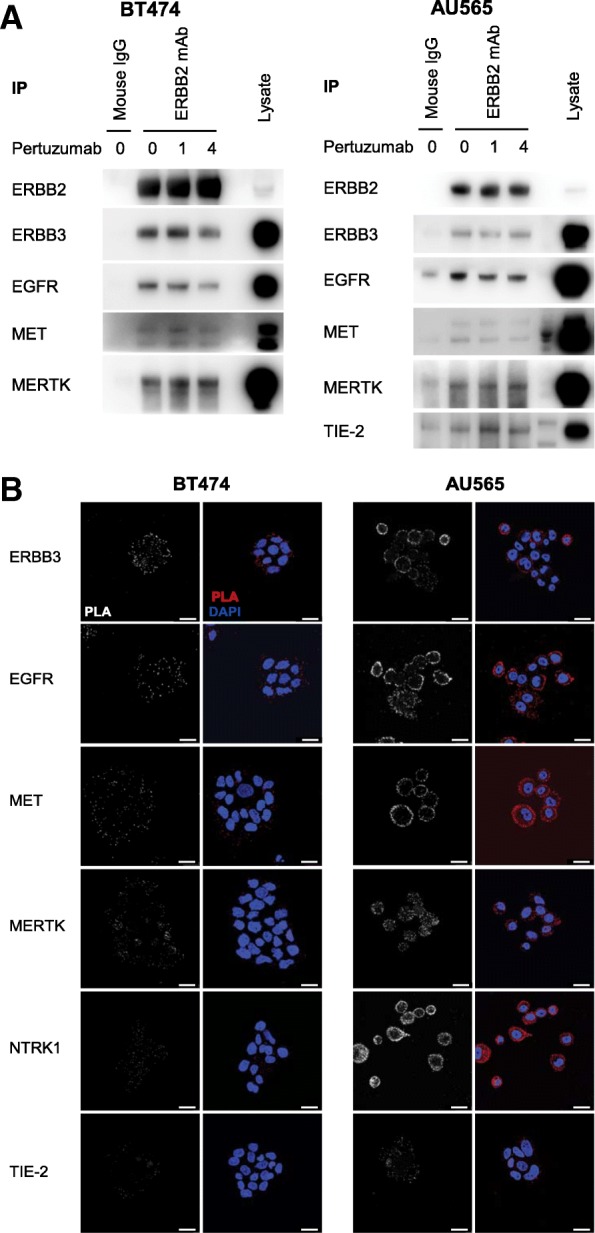
Receptor interactions with ERBB2. **a** Western blotting showing the co-immunoprecipitation of the indicated receptors with ERBB2 from BT474 cells and AU565 cells. Each line was treated with pertuzumab (100 nM) for the time period indicated, and immunoprecipitation was performed with either a mouse IgG control or ERBB2 monoclonal antibody. Blots are representative of three experiments. **b** Confocal fluorescence microscopy imaging of proximity mediated ligation assays showing the interaction between ERBB2 and the indicated RTKs in BT474 and AU565 cells (scale bar = 25 μm)

While pertuzumab increased the phosphorylation of this subset of non-canonical RTKs, it was not necessary to promote the physical interaction between ERBB2 and either MET or MERTK. Through co-immunoprecipitation, we could confirm that ERBB2 interacts with MET and MERTK in both BT474 and AU565 cell lines prior to exposure to pertuzumab (Fig. 4a). We also observed an interaction between the expected ERBB2 binding partners, EGFR and ERBB3, which decreased over time in the BT474 line. Interestingly, a robust interaction between TIE-2 and ERBB2 in the AU565 line was only observed following pertuzumab treatment (Fig. 4a), reflecting the absence of an interaction between these two receptors in our library screen (Fig. 2d).

While the interaction between ERBB2 and NTRK1 could not be observed by co-immunoprecipitation, the interaction between ERBB2 and EGFR, ERBB3, MET, MERTK and NTRK1 could all be observed by proximity-mediated ligation assay (PLA) in both the AU565 and BT474 cell lines (Fig. 4b, Additional file 2: Figure S5). The interaction between TIE-2 and ERBB2 could also be observed in the absence of pertuzumab using this method, albeit weakly.

### Pertuzumab blocks Akt but promotes ERK activation

**Fig. 5:**
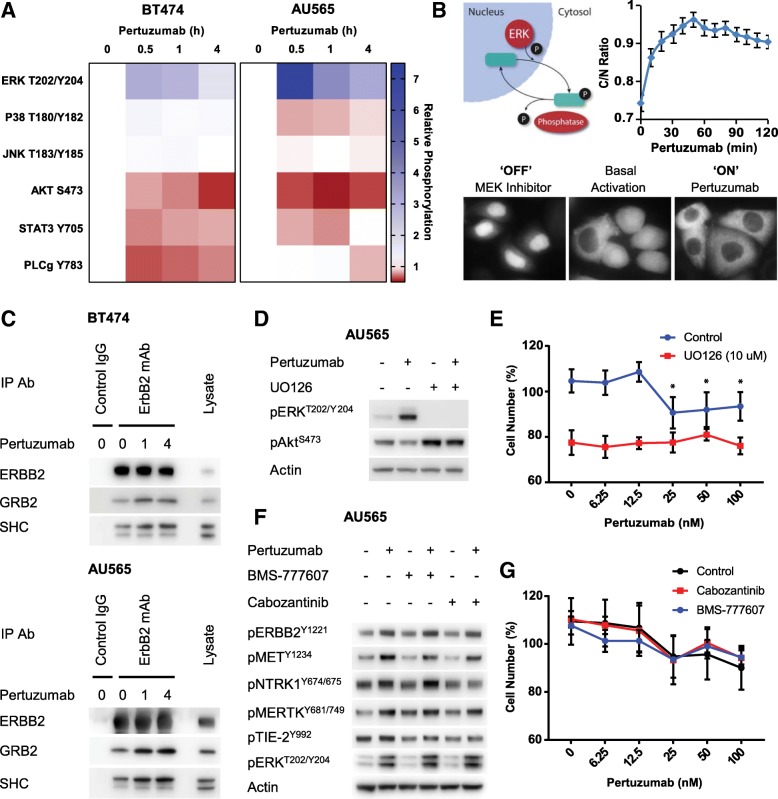
Targeting pertuzumab-induced signalling. **a** AU565 and BT474 cells were treated with pertuzumab (100 nM) for the time points indicated. Western blotting was performed with lysates from these cells with the total and phospho-antibodies indicated. Data is presented as a heat map showing the relative changes in phosphorylation (*n* = 3, mean); raw data and statistical analysis are presented in Additional file 2: Figure S6. **b** AU565 cells expressing the ERK-KTR clover biosensor were treated with pertuzumab (100 nM). Live cell imaging was performed to measure the cytoplasmic to nuclear (C/N) ratio of the ERK-KTR biosensor at the time points indicated (mean ± SEM). **c** Western blotting showing the co-immunoprecipitation of GRB2 and SHC with ERBB2 from BT474 cells and AU565 cells. Each line was treated with pertuzumab (100 nM) for the time period indicated and immunoprecipitation was performed with either a mouse IgG control or ERBB2 monoclonal antibody. Blots are representative of three experiments. **d** AU565 cells were treated with pertuzumab (100 nM) or UO126 (10 μM) for 30 min. Western blotting was performed with the antibodies indicated. Blots are representative of three experiments. **e** Cell viability assay was performed using the AU565 cell line with pertuzumab at the concentrations indicated, in the presence or absence of UO126 (10 μM) for 5 days (*n* = 6, mean ± SD, *p < 0.05). **f** AU565 cells were treated with the combination of pertuzumab (100 nM), BMS-777607 (1 μM) or cabozantinib (1 μM) for 30 min. Western blotting was performed with the antibodies indicated. Blots are representative of three experiments. **g** Cell viability assay was performed using the AU565 cell line with pertuzumab at the concentrations indicated, in the presence or absence of BMS-777607 (1 μM) or cabozantinib (1 μM) for 5 days (*n* = 6, mean ± SD)

Pertuzumab is known to effectively block ligand-induced signalling from ERBB2 to both Akt and ERK [35]. Therefore, to determine the downstream consequences of the paradoxical actions of pertuzumab on these different subsets of ERBB2-containing heterodimers, we performed western blotting for activation of the main RTK-mediated signalling pathways (Fig. 5a, Additional file 2: Figure S6). This analysis confirmed that pertuzumab treatment significantly decreased Akt phosphorylation in both the AU565 and BT474 cell lines, along with significantly decreased STAT3 phosphorylation in the AU565 line and PLCγ phosphorylation in the BT474 line. However, ERK phosphorylation was significantly increased at all time points of pertuzumab treatment in both cell lines. Notably, the pertuzumab sensitive cell line, ZR-75-30, displayed a weaker, transient activation of ERK in response to pertuzumab (Additional file 2: Figure S7A), which was only significant at the 30-min time point.

This ERK activation mediated by pertuzumab was also confirmed with an orthogonal, live-cell readout of ERK activation, the ERK-KTR biosensor (Fig. 5b). This biosensor is based upon the fluorescent protein Clover, fused to a phosphorylatable nuclear localisation sequence [36]. Upon phosphorylation by ERK, the biosensor shuttles out of the nucleus, allowing a readout of ERK activity based upon the cytoplasmic-to-nuclear (C/N) ratio of the fluorescent signal. By treating AU565 cells stably expressing the ERK-KTR biosensor with pertuzumab, we were able to monitor the C/N ratio over time by high-content imaging. This analysis demonstrated that following pertuzumab treatment, ERK activity increased with similar dynamics to that observed by the western blotting analysis (Fig. 5a).

In both the BT474 and AU565 cell lines, this pertuzumab-induced ERK activation was accompanied by increased interaction between ERBB2 and the receptor proximal, MAPK activating adaptor proteins, GRB2 and SHC (Fig. 5b). Hence, while pertuzumab can at least partially inhibit PI3K signalling through ERBB2:ERBB3 dimers, the interaction of ERBB2 with a subset of non-canonical RTKs promotes ERBB2 phosphorylation, recruitment of GRB2 and SHC and the downstream activation of ERK.

We therefore investigated whether direct inhibition of ERK activation using the MEK inhibitor UO126 would attenuate pertuzumab resistance within these resistant cell lines. While UO126 did prevent pertuzumab-induced ERK activation, it also elevated Akt activity and prevented the efficient inhibition of Akt by pertuzumab (Fig. 5c), in line with previous observations that MEK inhibition relieves negative feedback on ERBB receptors and thus promotes PI3K/Akt activation [37]. Accordingly, UO126 also displayed a weak inhibitory effect upon AU565 cell growth as a single agent, but not in combination with pertuzumab (Fig. 5d). Whereas in BT474 cells, UO126 did not influence cell growth as a single agent and completely abrogated any effect of pertuzumab (Additional file 2: Figure S8A).

### Targeting the network of non-canonical heterodimers

As the targeted inhibition of MEK/ERK was not a viable option, we next investigated whether targeting the network of non-canonical ERBB2 heterodimers would increase the efficacy of pertuzumab. We therefore utilised two broad-spectrum RTK inhibitors with nanomolar IC_50_ values for different combinations of these non-canonical RTKs. BMS-777607 is currently undergoing phase II clinical trials for a number of different cancers, including breast cancer, and has activity against MET (IC_50_ 3.9 nM), MERTK (IC_50_ 14 nM) and NTRK1 (IC_50_ 290 nM) [38]. Cabozantinib is also undergoing phase II clinical trials for breast cancer and has activity against MET (IC_50_ 1.3 nM) and TIE-2 (IC_50_ 14.3 nM) [39]. However, neither of these inhibitors could prevent pertuzumab-induced ERBB2 phosphorylation or ERK activation in AU565 cells (Fig. 5e). While cabozantinib did appear to prevent the pertuzumab-induced phosphorylation of TIE-2 and NTRK1, this was not sufficient to prevent ERK activation. In line with these observations, neither of these RTK inhibitors could increase the efficacy of pertuzumab in AU565 cells (Fig. 5f) nor BT474 cells (Additional file 2: Figure S8B).

As inhibition of these non-canonical RTKs did not prevent pertuzumab-induced ERBB2 phosphorylation, it is likely that the kinase domains of the non-canonical RTKs within these pertuzumab-activated heterodimers are acting as allosteric activators of the ERBB2 kinase domain. In this scenario, targeting these RTKs directly will not influence the signalling capacity of the heterodimer. Instead, targeting the ERBB2 kinase domain should prevent activation of both ERBB2 and all heterodimer combinations.

**Fig. 6:**
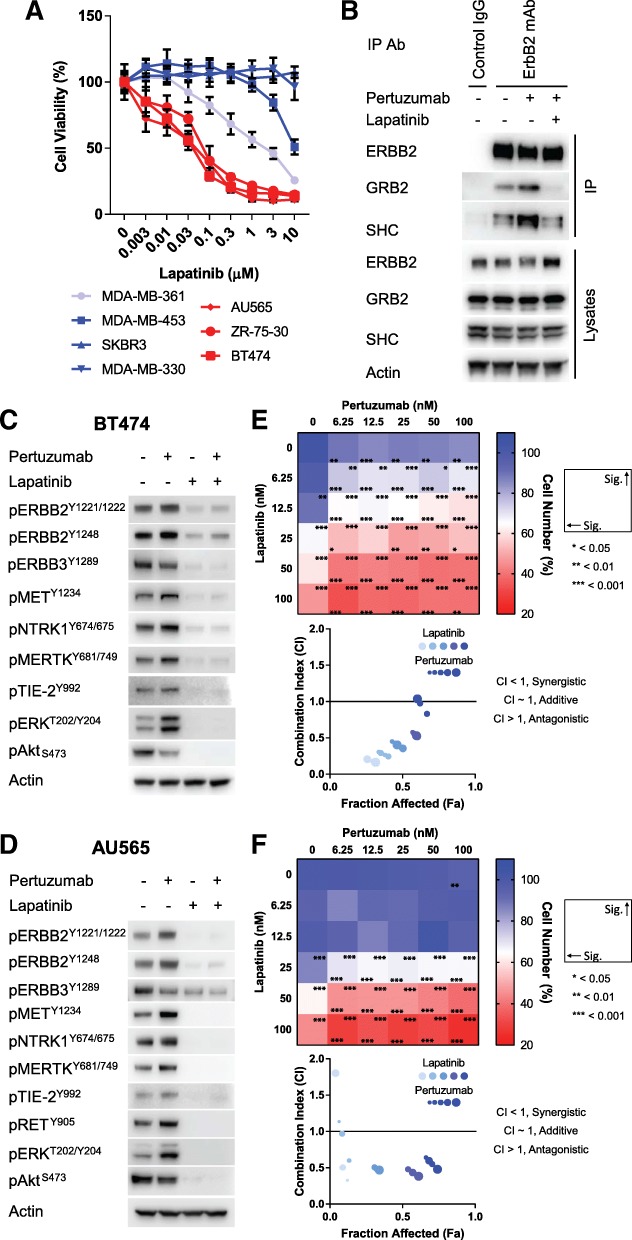
Synergy between pertuzumab and lapatinib in vitro. **a** Cell viability assays performed on a panel of ERBB2+ breast cancer lines with lapatinib at the concentrations indicated for 5 days (*n* = 6, mean ± SD). **b** Western blotting showing the co-immunoprecipitation of GRB2 and SHC with ERBB2 from AU565 cells treated with pertuzumab (100 nM) or lapatinib (100 nM) as indicated, for 30 min. Immunoprecipitation was performed with either a mouse IgG control or ERBB2 monoclonal antibody. Blots are representative of three experiments. **c** BT474 cells and **d** AU565 cells were treated with pertuzumab (100 nM) or lapatinib (100 nM) as indicated, for 30 min. Western blotting was performed with the antibodies indicated. Blots are representative of three experiments. **e** BT474 cells and **f** AU565 cells were used to perform combinatorial cytotoxicity assays with lapatinib and pertuzumab at the concentrations indicated for 5 days (*n* = 6, mean). Raw data is presented in Additional file 2: Figure S6C, D. Synergy was calculated as a Combination Index (CI), using Compusyn (v1)

Therefore, we investigated whether the TKI lapatinib was able to prevent the pertuzumab-induced activation of these non-canonical RTKs by ERBB2. In cell viability assays, lapatinib displayed strong activity as a single agent against all the high ERBB2-expressing ZR-75-30, BT474 and AU565 lines, with IC_50_ values between 50 and 100 nM (Fig. 6a). At higher concentrations, it was also effective against the lower ERBB2-expressing MDA-MB-361 and MDA-MB-453 lines, with IC_50_ values of ~ 1 μM and 10 μM, respectively.

In agreement with the hypothesis of allosteric activation, the treatment of BT474 cells with lapatinib prevented pertuzumab-induced recruitment of GRB2 and SHC to ERBB2 (Fig. 6b). Treatment of both BT474 and AU565 cells with lapatinib also decreased the activation of ERBB2, MET, NTRK1, MERTK, TIE2 and ERK under basal conditions and prevented their pertuzumab-induced activation (Fig. 6c, d). Lapatinib almost completely inhibited ERBB3 and AKT phosphorylation in the BT474 cell line (Fig. 6c), while the combination of lapatinib and pertuzumab had an additive effect upon ERBB3 and AKT phosphorylation in the AU565 cell line (Fig. 6d, Additional file 2: Figure S7B). Accordingly, lapatinib also increased the efficacy of pertuzumab in cell viability assays in both cell lines, resulting in a synergistic combination between these two ERBB2-targeting drugs (Fig. 6e, f, Additional file 2: Figure S8C, D).

Unlike the combination of pertuzumab and lapatinib, the combination of trastuzumab and lapatinib did not display any synergy in the AU565 cell line (Additional file 2: Figure S8E) nor did the combination of pertuzumab and trastuzumab (Fig. 1c). These findings suggest that within the setting of innate resistance driven by non-canonical ERBB2 heterodimers, only the combination of pertuzumab and lapatinib will give a beneficial combinatorial response.

It has been previously hypothesised that long-term treatment with lapatinib results in an adaptive response that rescues ERBB2 and ERBB3 activation, and consequently ERK and Akt signalling, through the increased expression of ERBB3 ligands [40]. After 24 h of lapatinib treatment in the AU565 cell line, we do observe a moderate recovery of ERBB3 phosphorylation and to a lesser extent Akt phosphorylation (Additional file 2: Figure S8F), but this is not influenced by the addition of pertuzumab. Instead, the effect of this drug combination on downstream signalling is already apparent at the earlier time points of drug treatment (Fig. 6c, d. Additional file 2: Figures S7B, S8F), and therefore, the synergy between these drugs does not result from long-term adaptation.

### In vivo confirmation of synergy

**Fig. 7:**
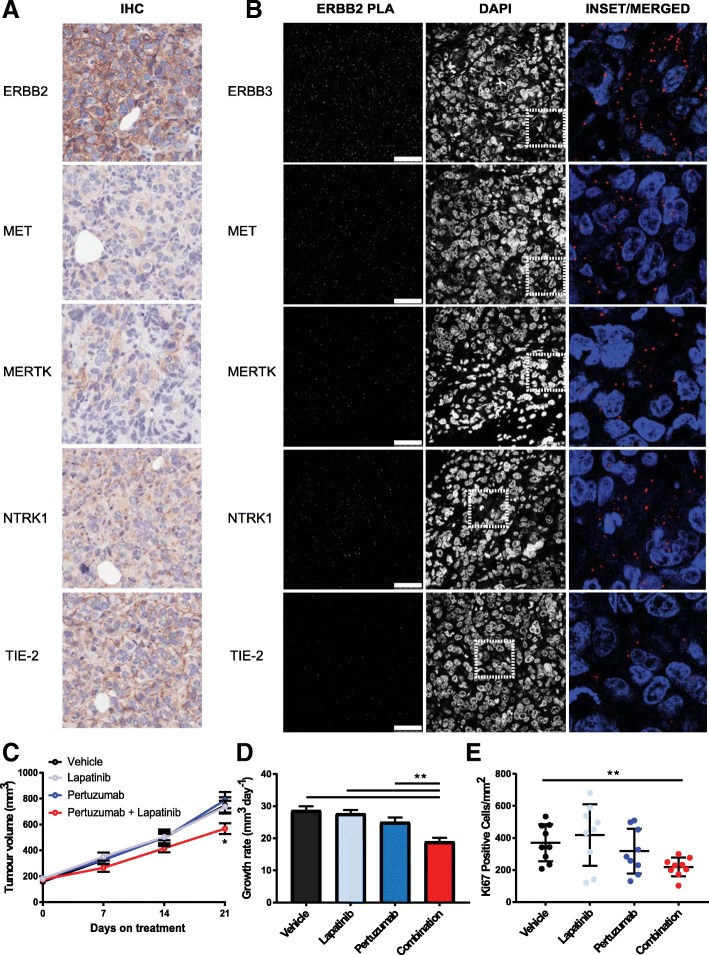
Synergy between pertuzumab and lapatinib in vivo. **a** IHC staining for RTK expression as indicated in the HCI-012 PDX model. (Scale bar = 25 μm). **b** Proximity mediated ligation assays detected heterodimers between ERBB2 and other RTKs as indicated, shown in red. (Scale bar = 25 μm). **c** Tumour growth following treatment with either vehicle, pertuzumab (12 mg/kg loading dose, 6 mg/k6 maintenance dose, intraperitoneal injection, once weekly), lapatinib (50 mg/kg, oral gavage, 5 times a week) or the combination of pertuzumab and lapatinib (*n* = 6, mean ± SEM, *p < 0.05). **d** Growth rate for each treatment arm calculated from a linear regression analysis (*n* = 6, mean ± SEM, **p < 0.01). **e** IHC staining for Ki67 from day 14 tumours. Analysis was performed on three random fields of view from three tumours for each arm (*n* = 9, mean ± SD, **p < 0.01)

To confirm the synergistic combination of pertuzumab and lapatinib within this resistant setting in vivo, we utilised the HCI-012 ERBB2+ PDX model, which originated from a patient who relapsed following treatment with trastuzumab and lapatinib [29] (Fig. 7). The expression of ERBB2 and the non-canonical RTKs MET, MERTK, NTRK1 and TIE-2 were observed by immuno-histochemistry within this PDX model (Fig. 7a, Additional file 2: Figure S9A). Furthermore, we also observed an interaction between ERBB2 and its canonical partner ERBB3, along with each of the non-canonical partners, using PLA (Fig. 7b, Additional file 2: Figure S9B).

Combination treatment with lapatinib and pertuzumab over 21 days significantly slowed tumour growth following sub-cutaneous implantation of this PDX within NOD-SCID-IL2γR−/− (NSG) mice (Fig. 7c, d). This response, albeit small, is remarkable given that this PDX originated from a trastuzumab and lapatinib resistant, relapsed tumour. In line with the patient treatment response, treatment with lapatinib alone did not result in a significantly slower growth rate, nor did single agent treatment with pertuzumab. Combination treatment also significantly decreased the expression of the proliferation maker Ki67 at the 14-day time point (Fig. 7e), indicating that only the combination of these two drugs reduced the growth rate of this otherwise single-agent-resistant ERBB2+ tumour.

## Discussion

Despite the improvements in overall and disease-free survival brought about by the introduction of ERBB2-targeting antibodies and inhibitors, there are still a large number of patients that either have no initial response or relapse following treatment with these drugs. This lack of response to ERBB2-targeting therapies in tumours with high ERBB2, and the lack of other markers of therapeutic response, highlights the need for a detailed mechanistic understanding of both the individual and combinatorial action of these drugs and the patho-physiological behaviour of this oncogenic receptor.

While it is commonly held that ligand binding induces dimerization of EGFR family members, a number of studies have identified pre-formed, ligand-independent dimers between a number of receptor pairings [31]. Many studies have also observed ligand-independent hetero-dimerization between EGFR family members and alternative RTKs, usually occurring under patho-physiological conditions in which one interacting partner is either amplified or highly expressed [21]. Our novel findings indicate that under the condition of ERBB2 amplification, ERBB2 forms a wide array of ligand-independent heterodimers both within and without the EGFR family.

From a structural perspective, it is unlikely that the extracellular domains of these receptors are mediating the observed non-canonical interactions, as the protein domain diversity present in these unrelated RTKs would be unlikely to support the formation of classical receptor dimers [13]. It is more likely that transmembrane or intracellular regions are facilitating these potentially low-affinity interactions. One potential example is the GXXXG motif present within the transmembrane regions of RTKs, which are thought to represent a general dimerization motif for transmembrane helices [41]. Notably, these motifs are not just present within EGFR family receptors, but are also present within a number of different RTKs [42], and are thought to promote a wide range of potential receptor interactions [43].

While non-canonical heterodimers are present in the absence of pertuzumab (with the notable exception of TIE-2), pertuzumab appears to act as an artificial ligand and potentially promotes a conformational change within the ERBB2 molecule that favours activation of these non-canonical receptor pairings. Detailed structural studies have revealed that following dimerization of EGFR family dimers, an asymmetric kinase domain dimer is formed, in which one kinase domain acts as an allosteric activator of the other kinase domain, which then performs trans- and auto-phosphorylation of the cytoplasmic tail of both receptors [44, 45]. Within our study, the inability of the broad spectrum RTK inhibitors BMS-777607 and cabozantinib to prevent pertuzumab-induced ERBB2 phosphorylation and ERK activation suggests that the kinase domains of these non-canonical RTKs are acting as allosteric activators of the ERBB2 kinase domain. This is also supported by the inhibition of MET, MERTK, NTRK1 and TIE-2 phosphorylation following treatment with lapatinib, suggesting that this phosphorylation is mediated by the ERBB2 kinase domain. This allosteric activation mechanism has previously been observed for the interaction of EGFR family members with non-canonical RTKs, including EGFR:PDGFRβ [46] and ERBB2:NTRK1 [47] heterodimers. In these studies, each EGFR family member underwent auto-phosphorylation following ligand-induced activation of the non-canonical dimerization partner, even in the presence of a specific kinase inhibitor for these receptors.

## Conclusion

Given the lack of efficacy for ERBB2-targeting drugs as single agents, significant focus has recently been placed on finding the most efficacious dual ERBB2-targeting regime [22]. The current standard of care for the treatment of metastatic ERBB2 breast cancer is the combination of pertuzumab, trastuzumab and a taxane in the first-line setting (7) and T-DM1 (Kadcyla, a trastuzumab-emtansine chemotherapy conjugate), in the second line setting [48]. We now demonstrate that the combination of pertuzumab and lapatinib represents a rationalised combination therapy based upon a mechanistic understanding of the patho-physiological formation of non-canonical ERBB2 heterodimers. The synergistic combination of these drugs in cell lines resistant to the combination of pertuzumab and trastuzumab, and the added benefit observed in a trastuzumab-resistant PDX model, suggests that further clinical investigation is warranted in patients that do not respond to either single antibody therapy or the combination of pertuzumab and trastuzumab. This could include patients such as those whose disease has progressed on the current standard first- and second-line metastatic therapy regimens, or patients who have progressed on trastuzumab-based combinations who are pertuzumab and lapatinib treatment naive. Currently, patients in this setting receive either a trastuzumab chemotherapy regimen or lapatinib in combination with capecitabine [49]. Based on our preclinical results, a clinical trial comparing pertuzumab with lapatinib with either of the currently used treatment combinations is warranted. Additionally, the investigation of these non-canonical RTKs (i.e. MET, MERTK, NTRK1 and TIE-2) as potential markers of response to the combination of pertuzumab and lapatinib should also be investigated.

## Additional files

Additional file 1: **Table S1.**. RTK plasmid library used for ERBB2 interaction screen. **Table S2.** Raw data for pertuzumab and lapatinib synergy assays. (DOCX 71 kb)

Additional file 2: **Figure S1.** RTK expression and ERBB2-targeting antibody response in breast cancer cell lines. **Figure S2.** Bimolecular fluorescence complementation based ERBB2 interaction screen. **Figure S3.** Tyrosine phosphorylation of ERBB2 following therapeutic antibody treatment. **Figure S4.** Analysis of RTK activation with phospho-specific antibodies. **Figure S5.** Control conditions for the ERBB2 proximity mediated ligation assay. **Figure S6.** Pertuzumab-induced signalling. **Figure S7.** Pertuzumab and lapatinib-induced signalling. **Figure S8.** Cell viability assays. **Figure S9.** Control conditions for IHC staining and PLA in breast cancer tissue. (PDF 883 kb)
