# Supplementary material for: Targeting promiscuous heterodimerization overcomes innate resistance to ERBB2 dimerization inhibitors in breast cancer
Source: Breast Cancer Res. 2019 Mar 21;21:43. doi: 10.1186/s13058-019-1127-y (PMC6429830; doi:10.1186/s13058-019-1127-y)

Additional file 1: Table S1 RTK plasmid library used for ERBB2 interaction screen.

| Gene Name | Destination Vector | Donor Vector Source | Plasmid ID | Stop Codon Removal |
| --- | --- | --- | --- | --- |
| ErbB2 | ORF-V1 | Addgene ORF | #23888 | NO |
| EGFR | ORF-V2 | Addgene ORF | #23935 | NO |
| ErbB2 | ORF-V2 | Addgene ORF | #23888 | NO |
| ErbB3 | ORF-V2 | Addgene ORF | #23874 | NO |
| ErbB4 | ORF-V2 | Addgene ORF | #23875 | NO |
| EPHA1 | ORF-V2 | Addgene ORF | #23929 | NO |
| EPHA2 | ORF-V2 | Addgene ORF | #23926 | NO |
| EPHA3 | ORF-V2 | Addgene ORF | #23911 | NO |
| EPHA4 | ORF-V2 | Addgene ORF | #23919 | NO |
| EPHA5 | ORF-V2 | Genecopoeia | GC-Z7535-CF | YES |
| EPHA8 | ORF-V2 | DNASU | HsCD00080451 | NO |
| EPHB1 | ORF-V2 | Addgene ORF | #23930 | NO |
| EPHB2 | ORF-V2 | DNASU | HsCD00080351 | NO |
| EPHB3 | ORF-V2 | Genecopoeia | GC-Z7536-CF | YES |
| EPHB4 | ORF-V2 | Addgene ORF | #23896 | NO |
| EPHB6 | ORF-V2 | Addgene ORF | #23931 | NO |
| FGFR1 | ORF-V2 | Addgene ORF | #23922 | NO |
| FGFR2 | ORF-V2 | Addgene ORF | #23904 | NO |
| FGFR3 | ORF-V2 | Addgene ORF | #23933 | NO |
| FGFR4 | ORF-V2 | Genomecube | OCAAo5051D1035D | YES |
| IGF1R | ORF-V2 | Genomecube | OCAAo5051C0674D | NO |
| INSR | ORF-V2 | Genecopoeia | GC-Y4566-CF | YES |
| INSRR | ORF-V2 | Addgene ORF | #23924 | NO |
| MST1R/RON | ORF-V2 | Addgene ORF | #23942 | NO |
| MET | ORF-V2 | Addgene ORF | #23889 | NO |
| CSF1R | ORF-V2 | Addgene ORF | #23928 | NO |
| PDGFRα | ORF-V2 | Addgene ORF | #23892 | NO |
| PDGFRβ | ORF-V2 | Addgene ORF | #23893 | NO |
| PTK7 | ORF-V2 | Genecopoeia | GC-A6381-CF | NO |
| RET | ORF-V2 | Addgene ORF | #23906 | NO |
| ROR1 | ORF-V2 | Genecopoeia | GC-A0499-CF | YES |
| ROR2 | ORF-V2 | Addgene ORF | #23927 | NO |
| RYK | ORF-V2 | Genomecube | OCABo5050A0218D | YES |
| TEK/TIE-2 | ORF-V2 | Genomecube | OCAAo5051D0794D | YES |
| NTRK1 | ORF-V2 | Addgene ORF | #23891 | NO |
| NTRK2 | ORF-V2 | Addgene ORF | #23883 | NO |
| NTRK3 | ORF-V2 | Addgene ORF | #23901 | NO |
| FLT1/VEGFR1 | ORF-V2 | Addgene ORF | #23912 | NO |
| FLT3 | ORF-V2 | Addgene ORF | #23985 | NO |
| FLT4/VEGFR3 | ORF-V2 | Addgene ORF | #23923 | NO |
| KDR/VEGFR2 | ORF-V2 | Addgene ORF | #23925 | NO |
| STYK1 | ORF-V2 | Addgene ORF | #23905 | NO |
| ALK | ORF-V2 | Addgene ORF | #23917 | NO |
| MERTK | ORF-V2 | Addgene ORF | #23900 | NO |
| TYRO3 | ORF-V2 | Addgene ORF | #23916 | NO |
| DDR1 | ORF-V2 | Addgene ORF | #23910 | NO |
| DDR2 | ORF-V2 | Addgene ORF | #23897 | NO |

**Table S2 Raw data for Pertuzumab and Lapatinib Synergy Assays.**


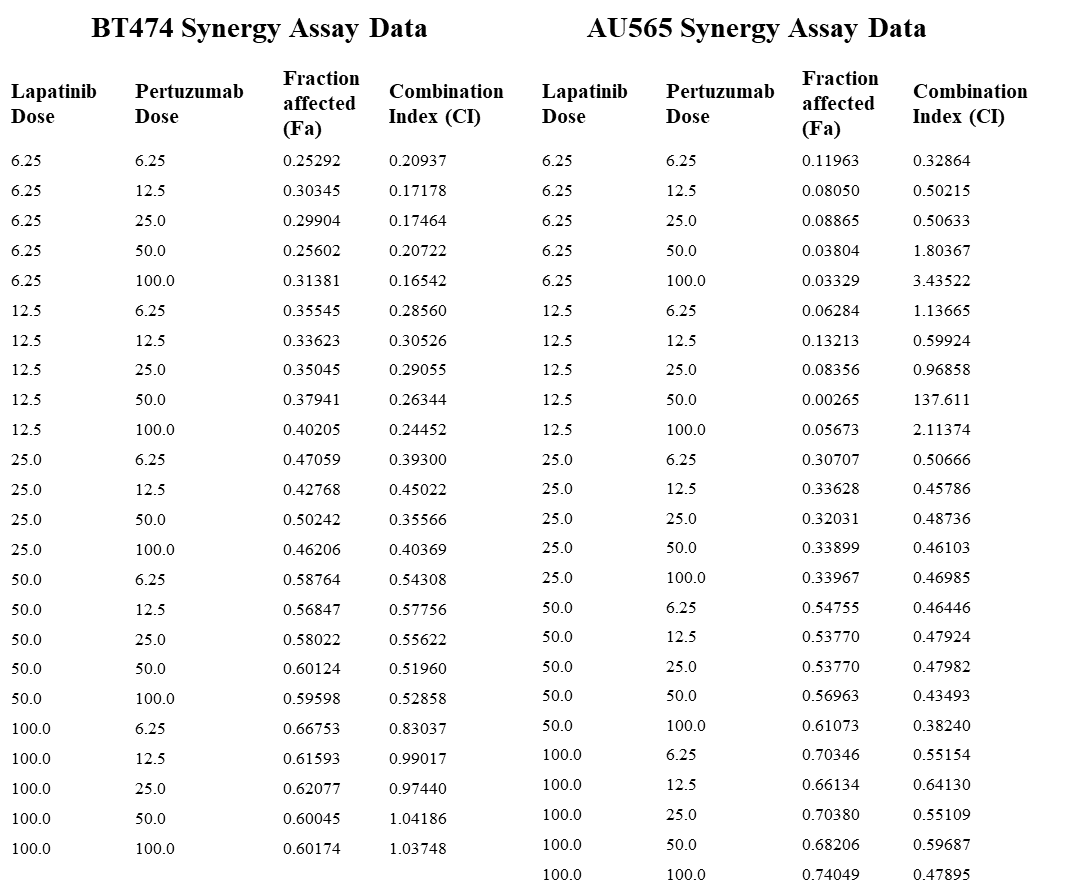

Supplement: Supplementary file 1 — Table S1.. RTK plasmid library used for ERBB2 interaction screen. Table S2. Raw data for pertuzumab and lapatinib synergy assays. (DOCX 71 kb) [file 13058_2019_1127_MOESM1_ESM.docx]
