# Supplementary material for: Targeting promiscuous heterodimerization overcomes innate resistance to ERBB2 dimerization inhibitors in breast cancer
Source: Breast Cancer Res. 2019 Mar 21;21:43. doi: 10.1186/s13058-019-1127-y (PMC6429830; doi:10.1186/s13058-019-1127-y)
Supplement: Supplementary file 2 — Figure S1. RTK expression and ERBB2-targeting antibody response in breast cancer cell lines. Figure S2. Bimolecular fluorescence complementation based ERBB2 interaction screen. Figure S3. Tyrosine phosphorylation of ERBB2 following therapeutic antibody treatment. Figure S4. Analysis of RTK activation with phospho-specific antibodies. Figure S5. Control conditions for the ERBB2 proximity mediated ligation assay. Figure S6. Pertuzumab-induced signalling. Figure S7. Pertuzumab and lapatinib-induced signalling. Figure S8. Cell viability assays. Figure S9. Control conditions for IHC staining and PLA in breast cancer tissue. (PDF 883 kb) [file 13058_2019_1127_MOESM2_ESM.pdf]

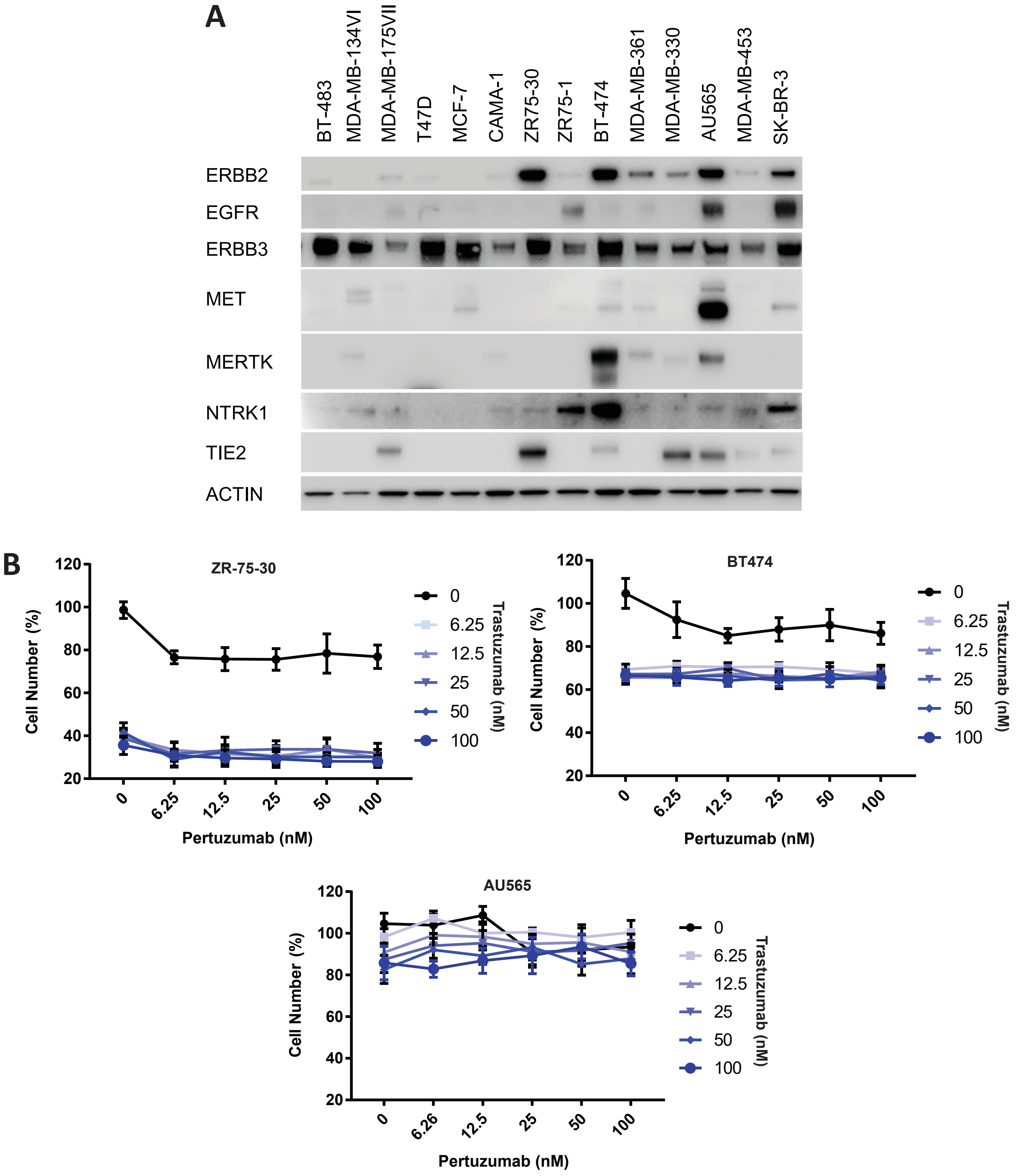

Figure S1: RTK expression and ERBB2 targeting antibody response in breast cancer cell lines. (**A**) Western blotting showing expression of the indicated RTKs in a panel of breast cancer cell lines. (**B**) Raw data for the combinatorial cell viability assays with Trastuzumab and Pertuzumab at the concentrations indicated for 5 days (n=6, mean ± SD).

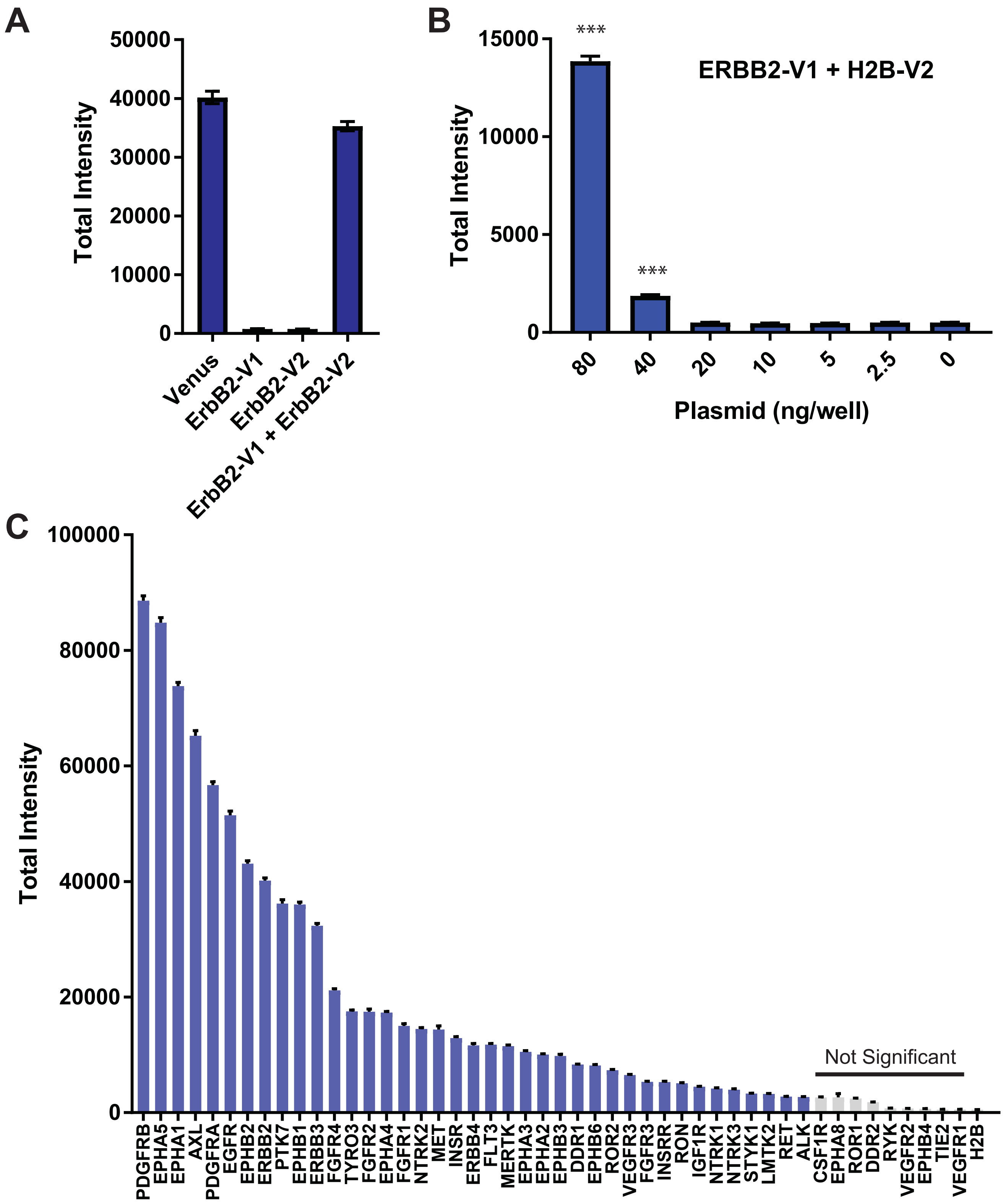

Figure S2: Bimolecular fluorescence complementation based ERBB2 interaction screen. (**A**) High content imaging analysis of fluorescence intensity in HEK-293T cells following transfection with plasmids containing a full length Venus control, ERBB2-V1, ERBB2-V2 or co-transfected with ERBB2-V1 and ERBB2-V2 (Mean ± SEM). (**B**) High content imaging analysis of fluorescence intensity in HEK-293T cells following transfection with decreasing amounts of plasmids containing ERBB2-V1 and H2B-V2 at a 1:1 ratio (Mean ± SEM). (**C**) High content imaging analysis of fluorescence intensity in HEK-293T cells following transfection with plasmids containing ERBB2-V1 (10 ng) and V2 tagged RTKs (10 ng), as indicated (Mean ± SEM).

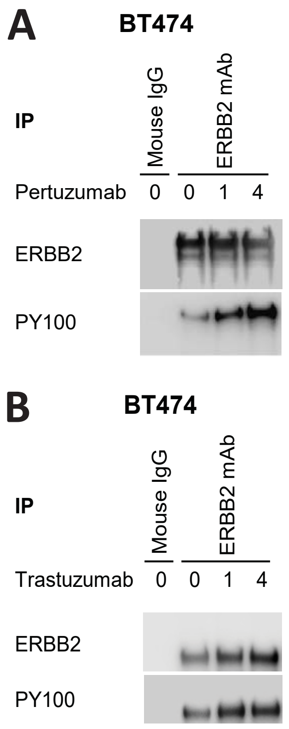

Figure S3: Tyrosine phosphorylation of ERBB2 following therapeutic antibody treatment. Western blotting showing the immunoprecipitation of ERBB2 from BT474 cells following treatment with (**A**) Pertuzumab (100 nM) or (**B**) Trastuzumab (100 nM) for the time period indicated. Immunoprecipitation was performed with either a mouse IgG control or ERBB2 monoclonal antibody, blotting was performed with PY100 and a rabbit ERBB2 antibody. Blots are representative of 3 experiments.

Kennedy et al. Figure S4

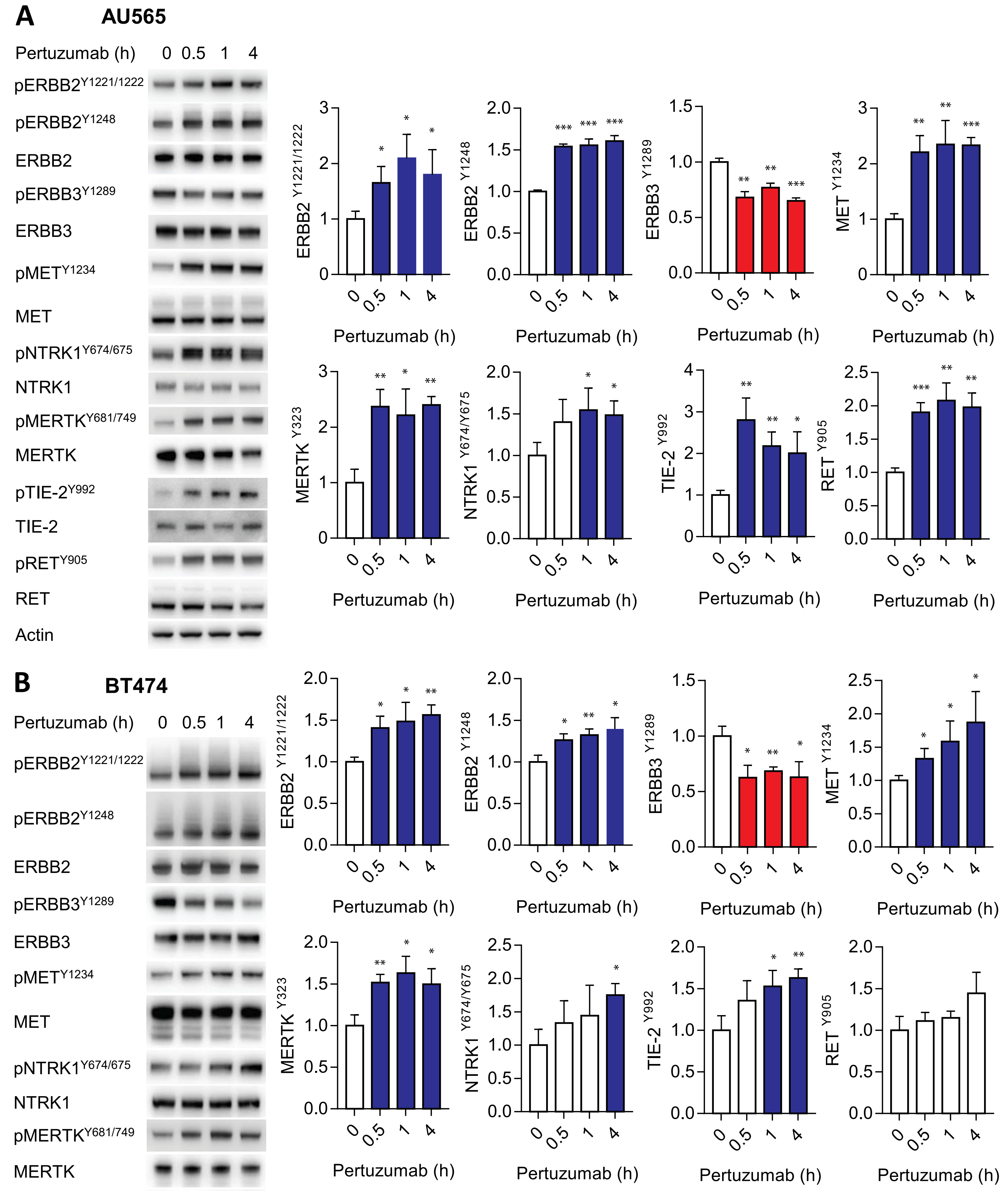

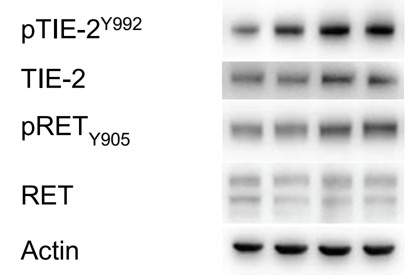

Figure S4: Analysis of RTK activation with phospho-specific antibodies. (**A**) AU565 cells, and (**B**) BT474 cells were treated with Pertuzumab (100 nM) for the time points indicated. Western blotting was performed with the total and phospho-RTK antibodies indicated. A representative image is shown from the three independent replicates used for quantification (n=3, mean ± SD, * p<0.05, ** p<0.01, *** p<0.001). Blue bars indicate significant upregulation, red bars indicate significant downregulation.

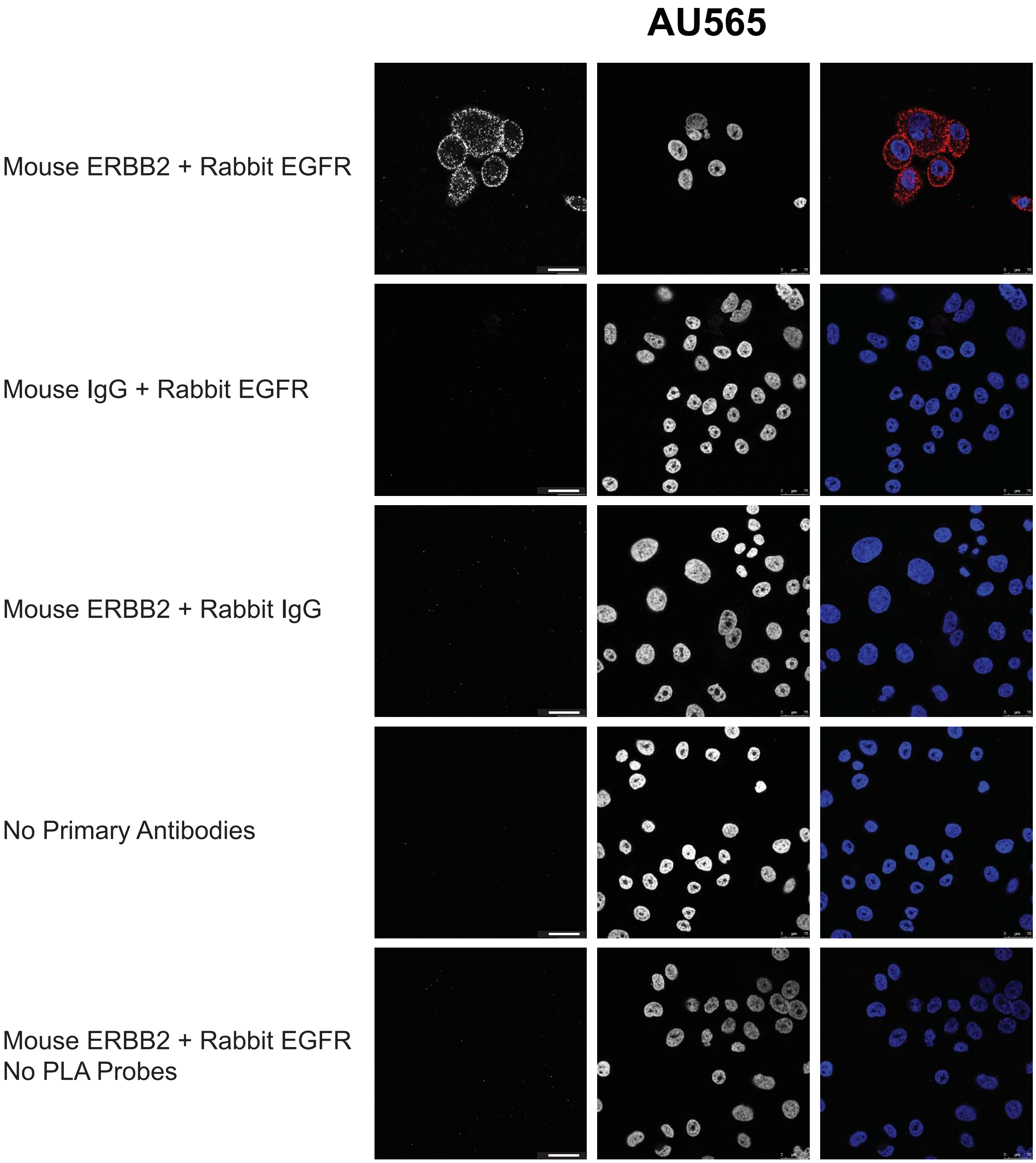

Figure S5: Control conditions for the ERBB2 proximity mediated ligation assay. Confocal fluorescence microscopy imaging of proximity mediated ligation assays showing the interaction between ERBB2 and EGFR in AU565 cells, and the indicated control conditions (Scale bar = 25 μm).

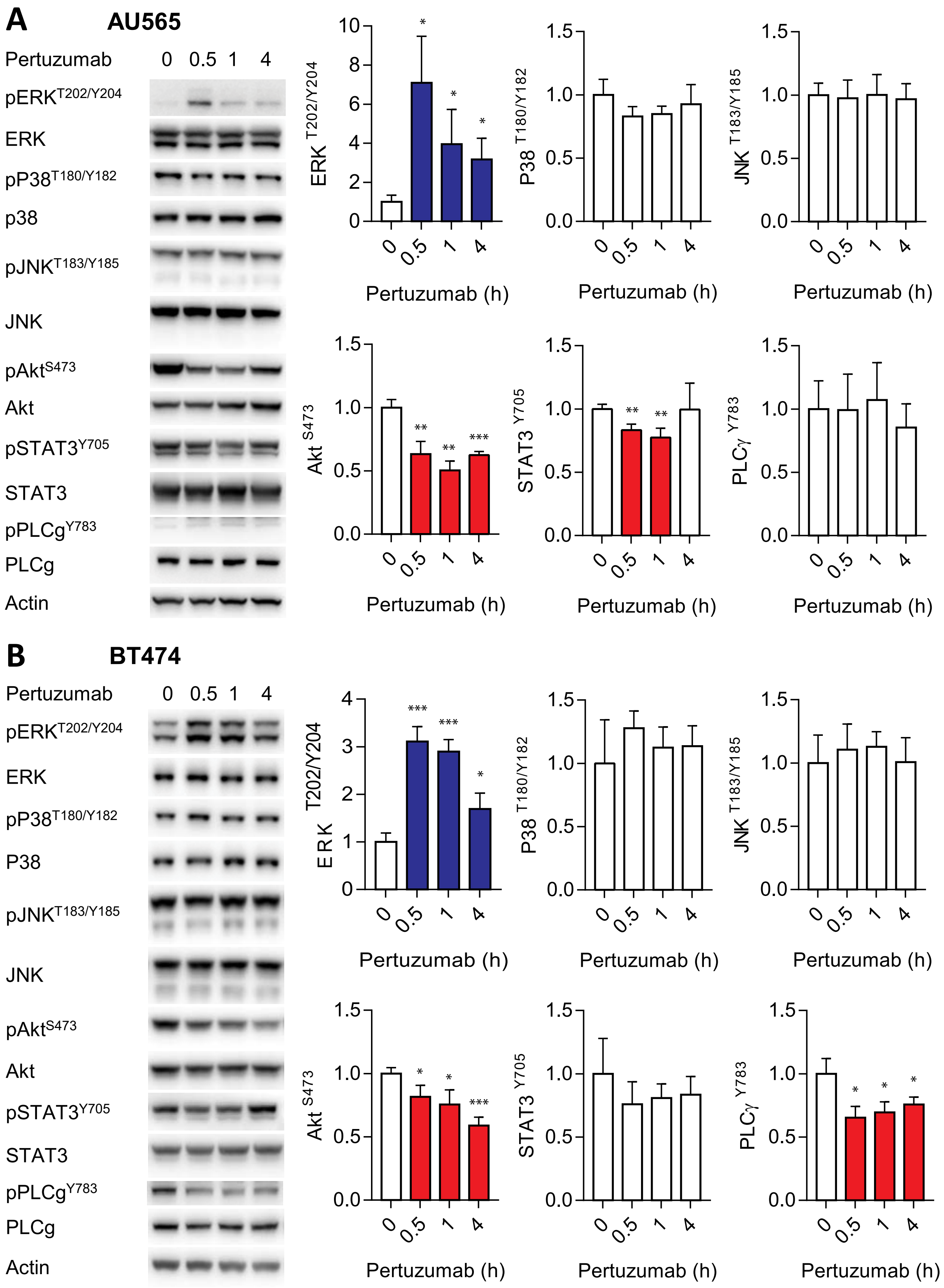

Figure S6: Pertuzumab induced signalling. (**A**) AU565 cells, and (**B**) BT474 cells were treated with Pertuzumab (100 nM) for the time points indicated. Western blotting was performed with lysates from these cells with the total and phospho-antibodies indicated. A representative image is shown from the three independent replicates used for quantification (n=3, mean ± SD, * $p<0.05$, ** $p<0.01$, *** $p<0.001$). Blue bars indicate significant upregulation, red bars indicate significant downregulation.

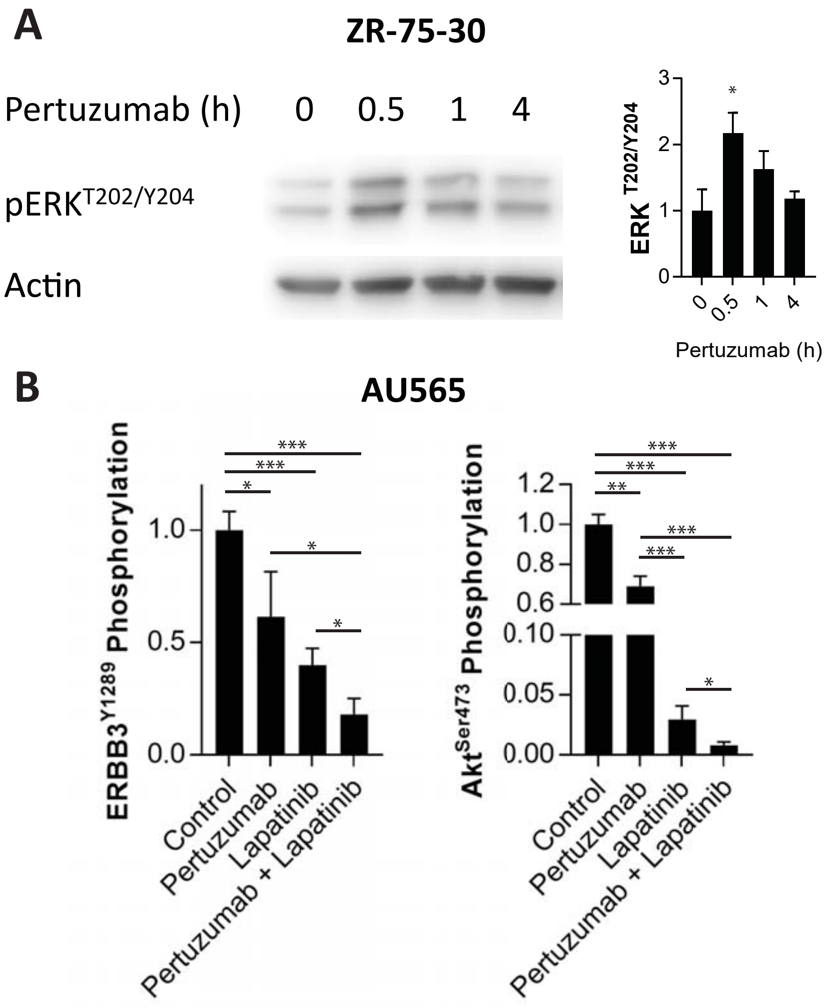

Figure S7: Pertuzumab and lapatinib induced signalling. (**A**) ZR-75-30 cells were treated with pertuzumab (100 nM) for the time points indicated. Western blotting was performed with lysates from these cells with the total and phospho-antibodies indicated. A representative image is shown from three independent replicates used for quantification. (**B**) Quantification of western blotting data from Figure 6D. AU565 cells were treated with pertuzumab (100 nM) or lapatinib (100 nM) as indicated, for 30 min. Western blotting was performed with the antibodies indicated. Quantification was performed on three independent replicates replicates (n=3, mean ± SD. * $p<0.05$, ** $p<0.01$, *** $p<0.001$).

Kennedy et al. Figure S8

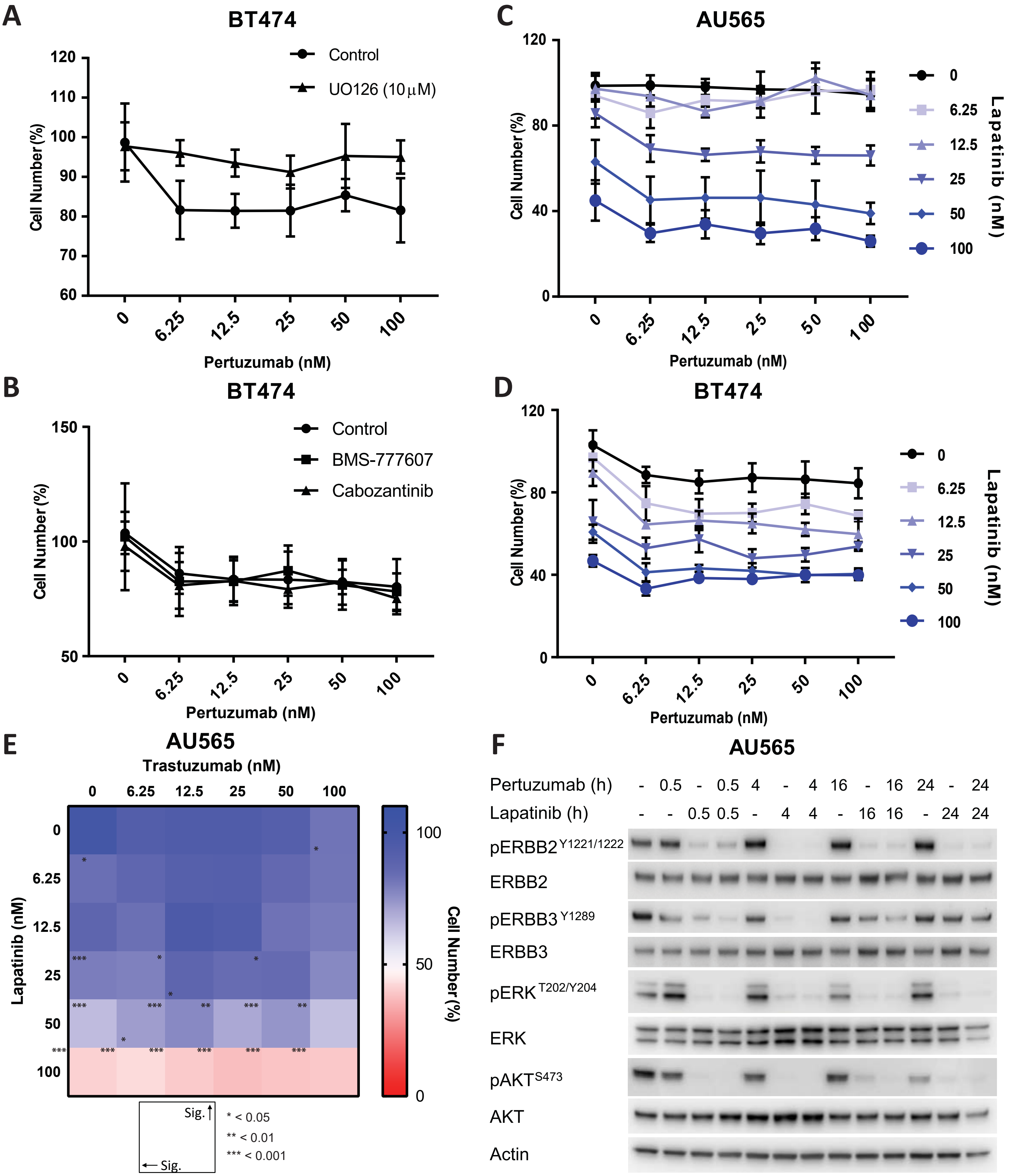

Figure S8: Cell viability assays. (**A**) Cell viability assay performed on the BT474 cell line using Pertuzumab and UO126 at the concentrations indicated for 5 days (n=6, mean ± SD). (**B**) Cell viability assay performed on the BT474 cell line using Pertuzumab, BMS-777607 and Cabozantinib at the concentrations indicated for 5 days (n=6, mean ± SD). (**C**) Raw data for the Pertuzumab and Lapatinib combinatorial cell viability assay performed on the AU565 cell line at the concentrations indicated for 5 day (n=6, mean ± SD). (**D**) Raw data for the Pertuzumab and Lapatinib combinatorial cell viability assay performed on the BT474 cell line at the concentrations indicated for 5 days (n=6, mean ± SD). (**E**) Combinatorial cell viability assay performed on the AU565 cell line using Trastuzumab and Lapatinib at the concentrations indicated for 5 days (n=6, mean ± SD). (**F**) Western blotting of the AU565 cell line following treatment with lapatinib (100 nM), pertuzumab (100 nM) or the combination of both, for the time points indicated.

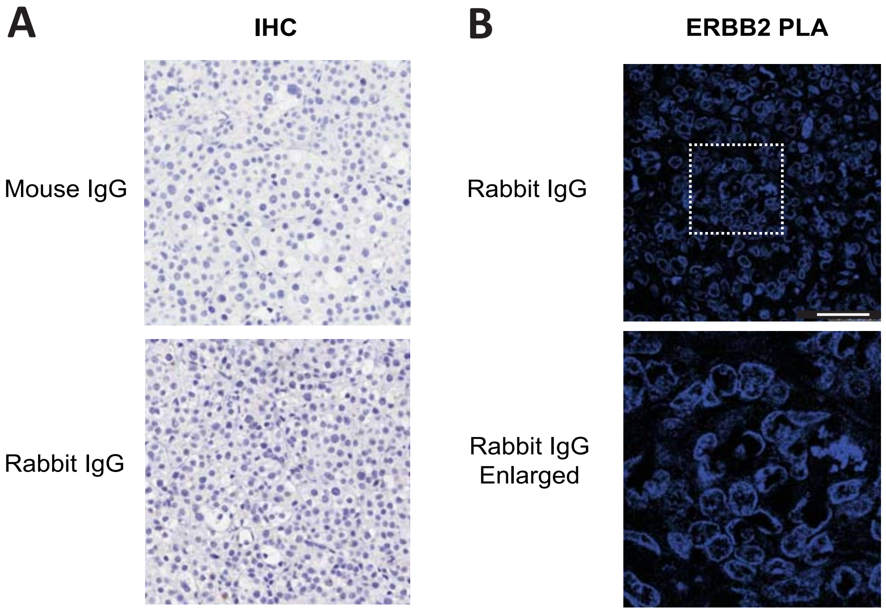

Figure S9: Control conditions for IHC staining and PLA in breast cancer tissue. (**A**) Control IHC staining with mouse and rabbit IgG in the HCI-012 PDX model. (**B**) Confocal fluorescence microscopy imaging of proximity mediated ligation assays in the HCI-012 PDX model showing the the indicated control conditions (Scale bar = 50 μm).
